# Tissue Extract Fractions from Starfish Undergoing Regeneration Promote Wound Healing and Lower Jaw Blastema Regeneration of Zebrafish

**DOI:** 10.1038/srep38693

**Published:** 2016-12-15

**Authors:** Yancen Dai, Nagarajan Prithiviraj, Jianhong Gan, Xin A. Zhang, Jizhou Yan

**Affiliations:** 1 Institute for Marine Biosystem and Neurosciences, Department of Biology, College of Fisheries and Life Sciences, Shanghai Ocean University, Shanghai 201306, China; 2 Key Laboratory of Exploration and Utilization of Aquatic Genetic Resources, Ministry of Education, Shanghai Ocean University, Shanghai 201306, China; 3 College of Food and Science, Shanghai Ocean University, Shanghai 201306, China; 4 Key Laboratory of Marine Biopharmaceuticals, Renji Hospital, Shanghai Jiaotong University School of Medicine, Shanghai, China; 5 Stephenson Cancer Center and Department of Physiology, The University of Oklahoma Health Sciences Center, OK 73104, USA

## Abstract

Natural bioactive materials provide an excellent pool of molecules for regenerative therapy. In the present study, we amputate portions of the arms of *Archaster typicus* starfish, extract and separate the active biomaterials, and compare the effects of each fraction on *in vitro* wound healing and *in vivo* lower jaw regeneration of zebrafish. Compared with crude extract, normal hexane fractions (NHFs) have a remarkable effect on cellular proliferation and collective migration, and exhibit fibroblast-like morphology, while methanol-water fractions (MWFs) increase cell size, cell-cell adhesion, and cell death. Relative to moderate mitochondrialand lysosomal aggregation in NHFs-cultured cells, MWFs-cultured cells contain more and bigger lysosomal accumulations and clump detachment. The *in vivo* zebrafish lower jaw regeneration model reveals that NHFs enhance blastema formation and vasculogenesis, while MWFs inhibit fibrogenesis and induce cellular transformation. Gene expression analyses indicate that NHFs and MWFs separately activate blastema-characteristic genes as well as those genes-related to autophagy, proteasome, and apoptosis either during cell scratch healing or ganciclovir-induced apoptosis. Our results suggest that bioactive compounds from NHFs and MWFs could induce blastema formation and remodeling, respectively, and prevent tissue overgrowth.

Tissue regeneration is an important regulatory phenomenon that has wide biological implications throughout the animal kingdom. Most vertebrate species and mammals, including humans, heal wound tissues with scar repair, whereas most invertebrates, such as sea stars, asteroidea, and planarians, can regenerate almost any parts of their bodies^1^. Among echinodermatous invertebrates, starfish with the typical deuterostomia-like developmental characteristics possess a striking repair capability. They can regenerate entire arms after both autotomic and traumatic amputation^2^. Unlike most vertebrates in which the regenerative capacity is generally limited to the healing of wounds, zebrafish can regenerate lost organs and restore complex tissue structures^3^,^4^,^5^.

The overall regenerative process has been studied in different asteroid species. The arm regeneration in *A. rollestoni* was achieved synchronously by *de novo* arm-bud formation and growth and arm-stump elongation^6^. In *E. sepositus*, the repair phase was characterized by prompt wound healing and re-epithelialization, followed by formation of a localized subepidermal edematous area^7^. Most studies suggest that regeneration in asteroids mainly relies on cell dedifferentiation, cell proliferation, cell migration, and the formation of blastema-like structures. In zebrafish, the regenerative process of the lower jaw involves epidermis reconstruction, blastema formation, and reformation. Blastema formation is a characteristic and transitional regeneration process. During restoration of the original skeletal structures, the blastema undergoes two phases of transitional transformation: mesenchymalization and chondrogenesis^4^. A zebrafish mutant (*dob*; devoid of blastema) cannot regenerate the fin, but has normal wound healing responses^8^. Thus, zebrafish and starfish might adopt different regenerative strategies to restore the lost structures at the phase of blastema formation.

Investigation into the regenerative mechanisms and processes in starfish and zebrafish can facilitate our understanding of how these animals overcome the specific selection pressure to enhance tissue regeneration. A related question of whether starfish regeneration is compatible with zebrafish regeneration merits further study. To address these questions, we now extend the use of the *in vitro* and *in vivo* model systems of zebrafish to the study of regenerative biomaterials.

Various starfish-derived bioactive substances, such as terpenes, sterols, cartenoid, astroponis, and phospholipase, have been identified to play important roles in anticancer therapy^9^. However, there is little information regarding tissue regeneration and the starfish-derived bioactive substances. For regenerative therapy, biomaterial cocktails and traditional medicines have exhibited regenerative and anti-inflammatory effects^10^. We therefore investigated the preparative isolation of active materials from the regenerating *A. typicus* starfish and evaluated their inhibitory and/or stimulatory effects on zebrafish cell lines and tissues. The present study offers an alternative, but promising, approach for future regenerative medicine.

## Materials and Methods

### Animals

Specimens of adult starfish *Archaster typicus*, ranging from 10–15 cm in overall diameter, were collected and maintained in aquaria containing aerated seawater at 25 °C with a timer-controlled light cycle with 14-h light and 10-h dark. Zebrafish were bred and maintained as previously described^4^. All experiments with zebrafish were performed in accordance with “Guide for the Care and Use of Laboratory Animals” (NIH), “Aquatic Veterinary Care and Animal Health in NICHD and Satellite Facilities”, and “Husbandry and Feeding of Zebrafish in the NHGRI”. All experimental protocols were approved by the laboratory animal care and use committee of Shanghai Ocean University.

### Tissue processing and histology

Starfish were anesthetized in 0.1% tricaine (3-aminobenzoic acid ethyl-ester methane sulphonate salt; Sigma, Poole, UK) in seawater. Approximately 0.5 cm of one arm-tip of each specimen was amputated. Starfish were further cultured for regeneration in aquaria for different periods. For histology observations on the regenerative processes, the regenerating tips of the arm stumps were re-cut at specific times, were fixed in Bouin’s fluid for 24 h, and were decalcified for a maximum of 24 h in 10% EDTA in filtered seawater^6^. Paraffin sections were prepared and stained with hematoxylin/eosin (HE) and other stains. Histological specimens from zebrafish were processed as previously reported^4^.

### Extraction and fractionation of bioactive materials

One arm-tip of about 1.5 cm was removed from each starfish specimen. Three hours later, the entire remaining starfish were processed for extraction and fractionation, as described in supplemental Fig. 1(Fig. S1). According to the method described elsewhere^11^, methanol water-soluble extracts were continually partitioned to give 5 fractions (WL_1–5_), called methanol water layer fractions. Meanwhile, normal hexane extracts were successively partitioned into 12 sub-fractions (NHL_1–12_), called N-hexane fractions. Collected fractions were lyophilized and stored at 4 °C. All of the isolated fractions were dissolved in dimethyl sulfoxide (DMSO) and were then subjected to further analyses.

### Cell culture and proliferation assay

PAC_2_, a zebrafish embryonic cell line, was gifted from Dr. Shawn Burgess’ laboratory. Cells were cultured in Leibovitz L15 medium supplemented with 15% fetal bovine serum and antibiotics^12^. Cell proliferation and cell viability was determined by MTT(3-[4,5-dimethyl thiazol-2-yl]-2, 5-diphenyl tetrazolium bromide) assay (MTT Cell Proliferation and Cytotoxicity Assay Kit, Sangong Biotech, Shanghai, China), which also revealed the integrity and activity of mitochondria, as described^13^. The PAC_2_ cells were seeded in 96-well tissue culture plates at a density of 1 × 10^5^ cells/ml. After reaching 80% confluence, the cells were incubated with different concentrations of the starfish tissue extracts (0.8, 4, 20, and 100 *μg/ml*) for 24 h. DMSO was used as a control. The attached cells were washed twice with phosphate buffer solution (PBS). Twenty *μl* of MTT stock solution (5 *mg/ml* in PBS) were added to each well, and the plates were further incubated overnight at 32 °C. One hundred *μl* of DMSO were added to each well to solubilize the formazan crystals produced by viable cells. After complete dissolution, the plates were agitated for 10 min, and absorbance was detected at 490 nm using a fluorometric ELISA plate reader (Spectramax, Gemini EM). This procedure was performed in triplicate in a parallel manner for each concentration.

### Mitochondria and lysosomal staining assay

Briefly, 1 × 10^5^ PAC_2_ cells were seeded in 96-well plates and were incubated with 100 *μg/ml* of *A. typicus* extract in each well for 24 hours. The same volume of DMSO was added to control wells. Then lysosome-(red dye) or mitochondria-(green dye) uptake solution (Mitochondrion Staining Kit and Lysosome Staining Kit, Sangon Biotech, China) was added to each well and was allowed to incubate for 1 hour. Thereafter, the supernatants were removed, DMSO was added, and incubation continued for 15 minutes. Fluorescence intensity was measured with a fluorometric ELISA plate reader (Spectramax, Gemini EM) at quantification of green (490 nm) and red (590 nm) fluorescence. All of the experiments were performed in triplicate. Inhibitor rate = (OD_control_ − OD_fraction_)/OD_control_ × 100%.

### Cell wound healing migration assay

The migratory behavior of PAC_2_ cells was analyzed using an *in vitro* cell wound-healing assay. Confluent cells (approximately 90%) or PAC_2_ monolayers were manually wounded by scraping the cells with a sterile P200 pipette tip. After removal of cellular debris by two washes of PBS, the wounded cells were cultured with fresh medium containing *A. typicus* extract (100 μg*/ml*), ganciclovir (GCV, Sigma; 1uM), or DMSO control. Wound healing was photographed with an Olympus digital camera attached to a light microscope. Cell migration was assessed by direct measurement of healed areas^14^.

### Zebrafish wound healing in *in vivo* and *ex vivo* assays

To provide a convenient and sensitive system for the quantitative evaluation of *in vitro* cell-level healing and *in vivo* tissue regeneration, gene expression profiling tests were designed. For the *in vitro* cell scratch healing assay, PAC_2_ cells were seeded onto Petri dish plates, were grown to 80–90% confluence, and were gently scratched with 200-μL pipette tips. The culture media were replenished at 24 h prior to RNA isolation. Cell cultures without any pretreatment were used as a control^15^.

For the *in vivo* wound-healing assay, adult zebrafish were anesthetized in 0.1% tricaine and one-third of the lower jaws were removed. The wounded fish were placed into tanks containing system water supplemented with 100 *μg/ml* of the bioactive fractions for 2 days. The regenerating jaws were collected for RNA isolation at 2 dpa and for histology analysis at 5 dpa^4^.

### RNA isolation and quantitative real-time PCR

Cells were harvested *via* trypsinization. Total cellular RNA was extracted with Trizol and used as a template to synthesize cDNA. Quantitative RT-PCR was performed in an Applied Biosystems cycler system following the manufacturer’s protocol. The gene expression level was analyzed by relative quantification, as previously described^16^. The primers used for qPCR are summarized in Supplemental Table 1.

### Statistical analysis

All experiments were carried out in triplicate and repeated at least twice. The differences among multiple sample groups (different treatments and concentrations) were analyzed by SPSS 16.0 using the Least-significant difference (LSD) *t*-test. Two-sample comparisons were also tested using the EXCEL two-tailed *t*-test. A level of *p* ≤ 0.05 was considered statistically significant.

## Results

### Tissue characterization of wound epidermis and arm regeneration of starfish

After amputation of the arm-tip (0.5 cm), the *Archaster typicus* regeneration rate was relatively slower than that reported in *Asterias Rolleston*bell^6^. As shown in Fig. S2, small outgrowth was seen on the sixth day. Regrowth of new arm-tips reached 2 mm in length on the 18th day. At 53 dpa, when regeneration was largely complete, the regenerated arms had increased an average of 4 mm.

**Figure 1:**
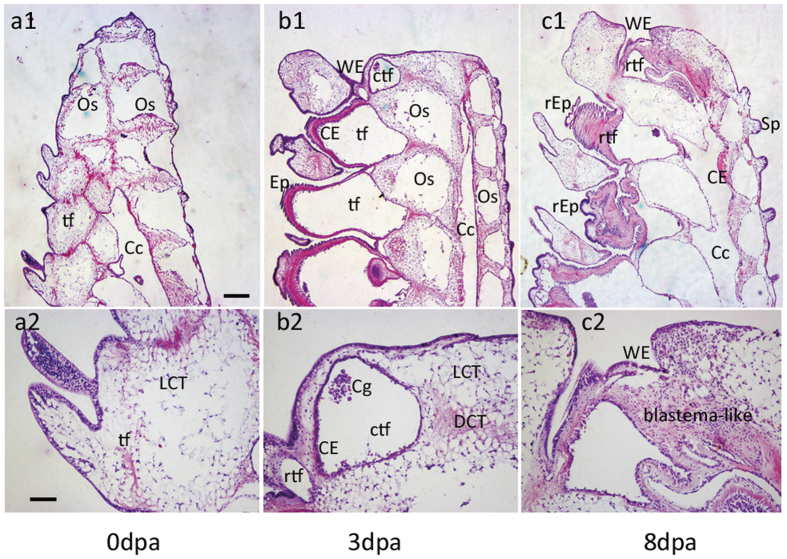
Anatomical features and histological processes in *A. typicus* arm regeneration. (**a**) Vertical sections of intact arm; (**b**) wound healing; (**c**) formation of blastema-like structure. **a2–c2** are magnifications of **a1–c1**, respectively. Cc, coelomic canal; CE, coelomic epithelium; Cg, coelomic granule elements; ctf, constricted tube foot or coelomic canal; DCT, dense connective tissue; Ep, epidermis; LCT, loose connective tissue; Os, ossicle; rEP, regenerating epidermis; rtf, regenerating tube foot; Sp, spine epidermis; tf, tube foot; WE, wound epidermis. Scale bars: 250 μm (**a1–c1**) and 50 μm (**a2–c2**).

Histologically, early regenerative processes were characterized by wound sealing, wound healing, and formation of blastema-like structures. Figure 1a shows the vertical sections of intact arm: tube feet with loose connective tissues, translucent liquid in coelomic cavity, and calcitic ossicles covering the coelomic canals. After amputation, the damaged arm stump and coelomic tube were strongly constricted. The epidermal cells around the tube feet near the wound site underwent proliferation, migrated into the wound, and sealed the wound. At 3 dpa, a sheet of the thick and organized wound epidermis completely covered the wound tissues and the constricted coelomic canal. The latter contained translucent liquid and coelomic granule elements (coelomic cells).The wound epidermis cells and adjacent epithelial cells from the coelomic canal increased and protruded into the dermis (Fig. 1b). By 8 dpa, a variety of epithelial cells and dermis cells aggregated toward the distal side of the coelomic canal, intermixed with dense connective tissue in a centripetal direction, and formed a blastema-like structure (Fig. 1c). Unlike the generic blastema of zebrafish^4^, no typical mesenchymal cells or dense extracellular matrix were observed.

### Starfish extract fractions have different bioactive effects on cell viability and cytotoxicity

**Figure 2:**
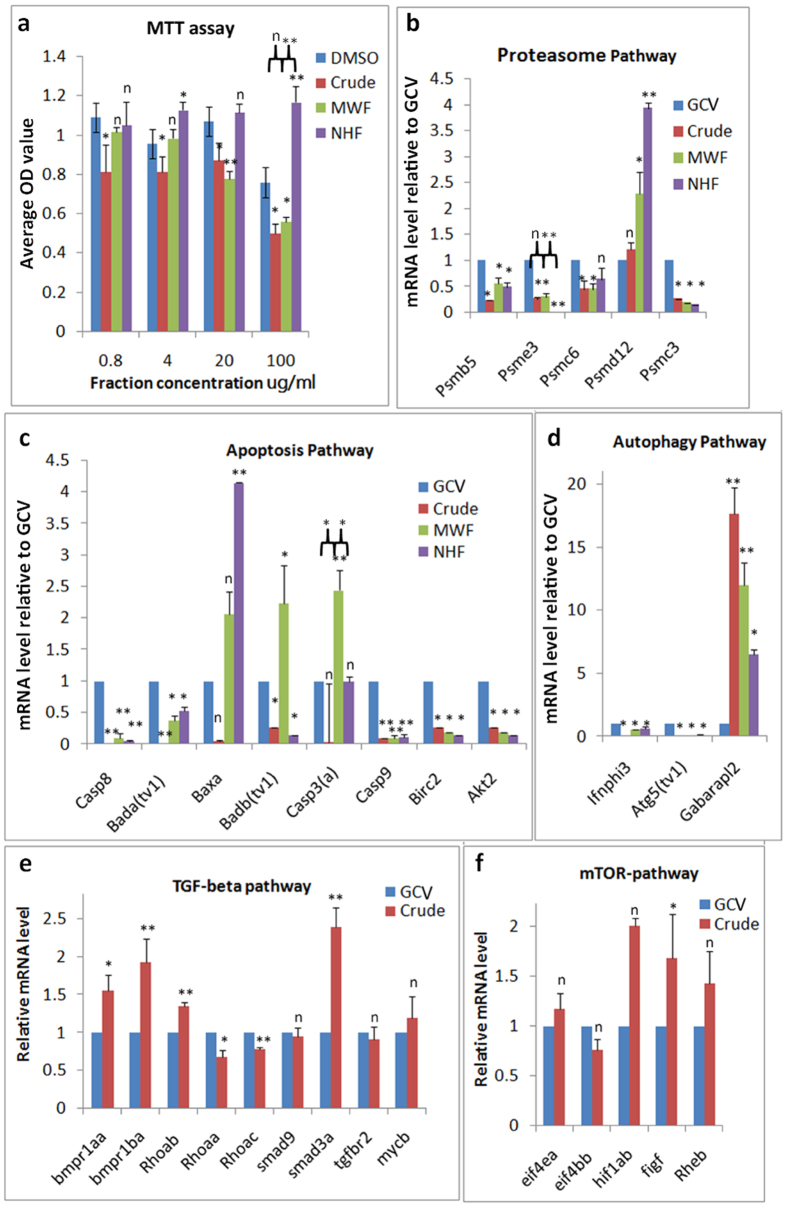
Evaluation of starfish extract fractions’ effect on cell growth and specific gene expression in PAC_2_ cells. (**a**) MTT assay; (**b**–**f**) quantification of gene expression relative to GCV treatment by real-time PCR. Statistical analyses: *P* > 0.05 (indicated by n); 0.01 < *P *< 0.05 (indicated by *); *P *< 0.01 (indicated by **). Significance between NHFs vs. MWFs, or MWFs vs. crude, is also labeled.

Blastema-like structures arise from the accumulation of a variety of cells at a specific region between the wound epidermis and damaged coelomic canal. This cluster behavior has also been observed during arm regeneration in the red starfish^7^. It is unclear whether the injured tissues or water vascular canal area release bioactive substances that direct cell growth and behavior. Because lower jaw amputation induces extensive responses in multiple zebrafish tissues as early as 2 hpa^5^ (data not shown), we cut the arm-tips to induce tissue regeneration, and then collected the entire remaining starfish for extraction. Cell viability and proliferation was determined with an MTT assay in the presence of extract fractions from *A. typicus*. The PAC_2_ cells were cultured and treated with various concentrations of extracts (0.8–100 *μg/ml*) for 24 h. Based on similar viability and cytotoxicity indicated by the highest OD value, we pooled the water-soluble methanol fractions (OD _WL1–4_ = 0.53–0.66), termed MWFs, and the n-hexane fractions (OD_NHL3–7_ = 1.1–1.4), termed NHFs (Fig. S1). As depicted in Fig. 2a, the starfish extracts generally produced a dose-dependent increase in cell viability in the crude and NHFs, and a dose-dependent decrease in MWFs at doses from 0.8 *μg/ml* to 20* μg/ml*. However, these dose-response trends changed at 100* μg/ml* of starfish extract. Compared with control (DMSO), MWFs and crude extract showed 26.5% and 34.1% inhibition of cell viability, while NHFs significantly promoted cell proliferation and viability by 54%. Under the microscope, PAC_2_ cells exposed to 100* μg/ml* concentrations of biomaterial fractions became more easily detached and suspended in MWFs medium than did those in NHFs. The control group cells maintained their usual morphology and grew into confluent monolayers.

### Extract fractions alter PAC2 cell phenotype and behavior

**Figure 3:**
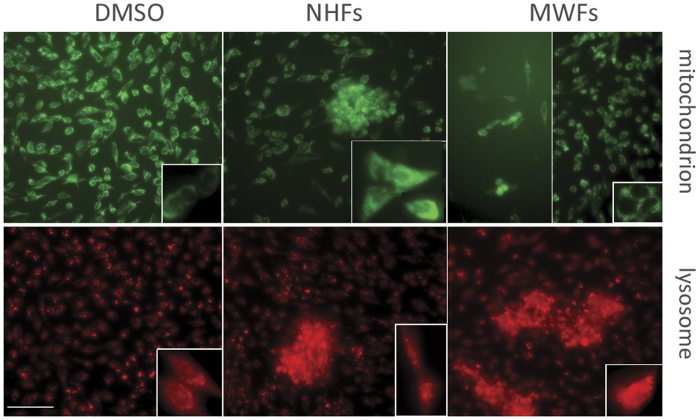
Fluorescence images of mitochondrion and lysosome staining. PAC_2_ cellswere exposed to the extracts (100 *μg/ml*) or the same volume of DMSO. (**a**) DMSO control, (**b**) NHFs treatment, (**c**) MWFs treatment. Mitochondria and lysosomes are stained green and red, respectively. Insets show magnification of individual cells. Scalebar: 50 μm.

To test whether our isolated bioactive materials affect lysosomal and mitochondrial biogenesis in PAC_2_ cells, we performed double staining of these two organelles using LysoTracker (red) and MitoTracker (green) probes. Unlike the uniform monolayer distribution of cells and fluorescence signals observed in DMSO controls, both NHFs and MWFs showed adherent cell aggregates (cell patches like) of different sizes. Compared with NHFs, MWFs treatment caused more lysosome clustering and fusion, and less mitochondrial aggregation. Consistent with the reduced cell viability shown by MTT assay, MWFs caused cell detachments, giving rise to cell-sparse zones (Fig. 3). Thus, mitochondrial and lysosomal staining analyses revealed that NHFs and MWFs differentially enhanced cell aggregation as well as mitochondria and lysosome accumulations.

### Extract fractions modulate cell wound healing

The lysosome and the mitochondrion play crucial roles in apoptosis- and autophagy-mediated cell growth and death^17^. Ganciclovir (GCV) is often used in anticancer gene therapy, as it kills cells and induces apoptosis^18^. To investigate the potential effect of *A. typicus* extracts on wound healing and cell apoptosis, an *in vitro* PAC_2_ cell wound-healing assay was performed, using GCV as an apoptosis control. In Figure S3a, representative images of wound healing show that cell migration increased by 11.6% in NHFs-treated cells and by 49.8% in GCV-treated cells, compared with the cell movement of the DMSO control and crude extraction groups. In contrast, cell migration in cells receiving MWFs treatment was inhibited up to 14.6% (Fig. S3b). To better characterize the changes in cell migration, we compared cell density and cell morphology at 48 h after scratching (Fig. S3a). We found that GCV-treated cells appeared eutrophic and crowed, with many floating cells. NHFs cells exhibited a slender, fibroblast-like morphology, and collective migratory behaviors. MWFs-treated cells appeared to be hypertrophic and formed clumps, which further coalesced into larger cell aggregates. DMSO- and crude extraction-treated cells did not show such distinct phenotypes and directional migrations. These results suggest that *A. typicus* extract fractions and GCV act on cell proliferation and behaviors in different ways.

### Extract fractions differentially activate the apoptosis, proteasome, and autophagy pathways

To unveil the molecular mechanism underlying cellular phenotypic changes caused by *A. typicus* extract fractions, we analyzed the genes related to apoptosis, autophagy, and the proteasome (Fig. 2b–d). These genes (supplemental Table 1) have been previously found to be deregulated during zebrafish lower jaw regeneration^5^. Compared with GCV treatment, two genes were simultaneously induced by all *A. typicus* extract fractions. These included the highest expression of *psmd12* in NHFs, and maximally activated *gabarapl2* in the crude extract. Because three apoptosis-related genes, *baxa, badb*(*tv1*), and *casp3a*, were upregulated, and two GCV-induced apoptosis-related genes, *casp8* and *casp9*, were downregulated, we concluded that MWFs induced different and incomplete apoptosis responses.

Since NHFs and MWFs displayed an overlapping, but distinct, gene expression profile, and the crude extract expectedly reflected mild and integrative responses of NHFs and MWFs bioactivities, we then tested which factors mediate the apoptosis and autophagy responses to the crude extract. TGF-beta (transforming growth factor-beta) signaling systems acting as major growth regulatory pathways crosstalk with mTOR-autophagy genes and apoptosis genes to control cell growth and death^19^,^20^. In response to the crude extract vs. GCV, six genes related to the TGF-beta were deregulated, including four upregulated genes (*bmpr1aa, bmpr1ba, smad3a*, and *Rhoab*) and two downregulated genes (*Rhoaa* and *Rhoac*). Only one mTOR pathway gene (*figf*) was activated (Fig. 2e,f). Combined with downregulation of all apoptosis genes and moderate autophagy responses to the crude extract, this result suggests that the integrative responses in the starfish extract-treated cells might be modulated through a central regulatory mechanism of the TGF-beta signaling system.

### The proteasome-autophagy signaling axis dominates in cell scratch healing

**Figure 4:**
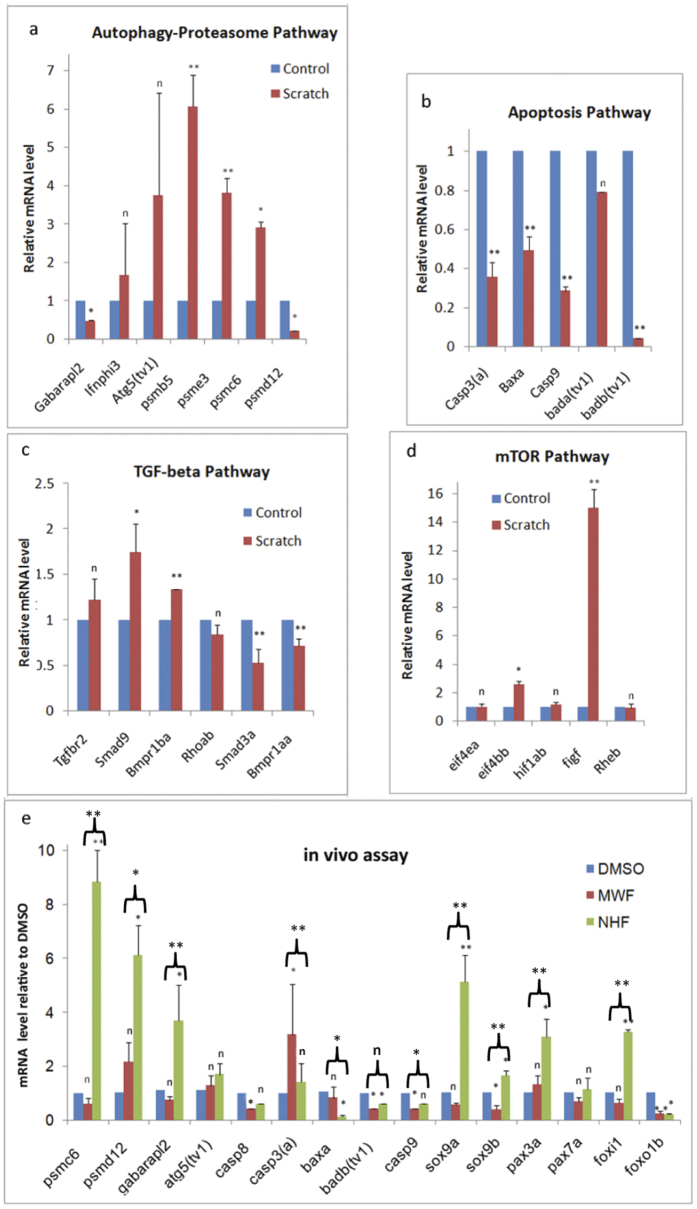
Analyses of gene expression in response to wound healing. (**a**–**d**) Show PAC_2_ cell wound healing, as determined by *in vitro* scratch assay; (**e**) shows lower jaw wound healing at 2 dpa. The autophagy-proteasome pathway genes were largely activated (**a**), and all tested apoptosis genes were down regulated (**b**). Further, the genes associated with the mTOR and TGF-beta pathways were selectively activated (**c**,**d**). (**e**) Effects of MWFs and NHFs on gene expression during zebrafish wound healing at 2 days after amputation of the lower jaw. Same statistical analyses as in Fig. 2. Significance between NHFs vs. MWFs is also labeled.

In order to determine whether the above autophagy and apoptosis mechanisms act in cell-level healing, we extensively scratched the PAC_2_ cell surfaces and then examined their gene responses. The genes associated with the proteasome and autophagy, such as *psme5, psme3*, and *psmc6*, were markedly upregulated. Conversely, *gabarapl2, psmd12*, and all tested apoptosis genes were downregulated (Fig. 4a,b). Compared with levels in the intact monolayer, the levels of mTOR genes *figf* and *eif4bb* were increased by 14.9 and 2.5 folds, respectively, in the wounded monolayer (Fig. 4c). Meanwhile, *smad9, bmpr1ba, bmpr1aa*, and *smad3a* in the TGF-beta pathway were deregulated (Fig. 4d). This result indicated that cell scratch healing activates the autophagy and proteasome pathways, but inhibits the apoptosis pathway, possibly through TGF-beta and mTOR signaling regulation. Since our cell scratch healing experiment involved only a single type of cell line, we suspect that those proteasome-autophagy-related genes were induced to facilitate cell proliferation.

### Starfish biomaterials modulate zebrafish lower jaw regeneration

**Figure 5:**
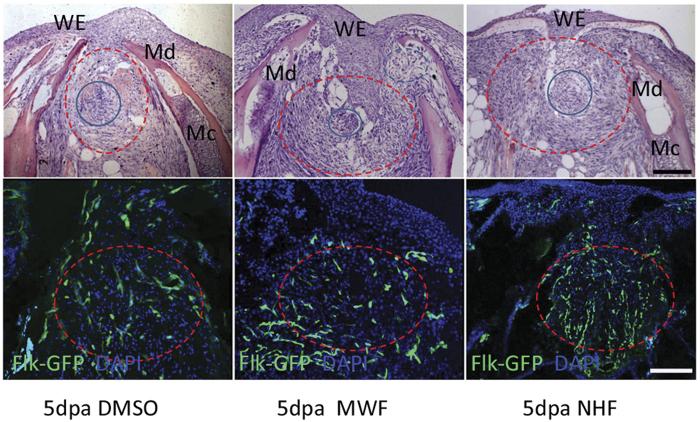
Starfish biomaterials affect wound epidermis reconstitution and blastema formation of the zebrafish lower jaw at 5 dpa. The upper row shows HE staining, while the bottom row shows the Flk-GFP signal in flk1 promoter-driven GFP transgenic zebrafish. The dotted red line demarcates the blastema region. The blue circle indicates cell clusters or presumed chondrogenic centers. The dotted blue circle shows chondrocytes. WE, wound epidermis; Md, mandible; Mc, Meckel’s cartilage. Scale bars: 100 μm.

After discovering that starfish extract fractions differentially activated the gene pathways that regulate cell phenotypes, we then investigated the efficacy of these active materials on the tissue regeneration of the zebrafish lower jaw (Fig. 5). We cultured the zebrafish in system water containing the biomaterials for 2 days, and examined the histological structures of the regenerating lower jaw at 5 dpa.

As previously reported^5^, complete reepitheliation and transition from mesenchymal blastema to chondrogenic blastema were clearly observed in DMSO control specimens (Fig. 5). A similar regeneration process took place in jaws treated with NHFs, although more blastema cells (fibroblast-like) with tissue-specific orientation and more Flk-GFP-labeled blood vessels were observed. Clearly, the blastema formation was positively correlated with blastema vasculogenesis, as indicated by Flk-GFP signal intensity. However, MWFs-treated jaws showed a thicker stratified epidermis, fewer fibroblast-like cells and matrix, and more disorganized chondrocytes and myofibrils. It appeared that MWFs decreased blastema fibrogenesis, but increased muscular and chondrogenic differentiation. The results consistently indicated that NHFs enhanced fibroblast-like cell proliferation and directional migration, while MWFs induced cytotoxic responses and cellular transformation.

The gene expression analyses at 2 dpa showed that NHFs preferentially upregulated proteasome and autophagy genes (*psmc6, psmd12*, and *gabarapl2*), while MWFs highly upregulated *caps3a* and downregulated *caps8* and *casp9* (Fig. 4e). Five tissue-specific genes, *flk1, pax3a, sox9a, sox9b*, and *foxi1*, were upregulated by NHFs, although *pax3a* was also slightly activated by MWFs. These genes are characteristically expressed in blastema^5^, and therefore were used as blastema characteristic marker genes. Together with the gene expression profile shown in Figs 2b–f and 4, the proteasome gene (*psmdl2*), autophagy gene (*gabarapl2*), and apoptosis genes (*bax3a, badb*(*tv1*), *casp3a*) were conformably activated by at least one of the three types of starfish extracts. However, these genes were totally suppressed in PAC_2_ cell scratch healing. These results suggest that our starfish extracts contain bioactive compounds to regulate the activities of *psmdl2, gabarapl2, and casp3a*; their activities play crucial roles in complex tissue regeneration.

## Discussion

Construction of three-dimensional neotissues requires the combined use of cells, engineering materials, and suitable biochemical and physicochemical factors to improve biological tissues. One such improvement is to establish bioactive factors or biomaterials from the species that have high tissue regeneration capabilities. The striking ability of starfish echinoderms to regenerate their injured organs and lost body parts is a valuable experimental model for studying all aspects of regenerative processes, from molecular to macroscopic levels^21^. The present study was intended to investigate the possibility and feasibility of the application of starfish biomaterials to regenerative medicine.

First, we address the extent to which starfish arm regeneration differs from zebrafish regeneration. In addition to blastema structure, wound sealing, wound healing, wound epidermis reconstitution, and epithelial/mesenchymal interconversions represent the basic processes of nearly complete healing in both zebrafish and starfish^2^,^7^,^22^. In contrast to zebrafish blastema, which is composed of a mass of undifferentiated mesenchymal cells with excessive extracellular matrices, starfish blastema-like structures are more likely to be an accumulation of proliferating cells in matrix and lack typical fibroblast features. According to traditional views, regeneration involves two alternative basic mechanisms: epimorphosis and morphallaxis. In epimorphos, new tissues arise from undifferentiated cells (stem cells or de-differentiated cells) and a typical blastema formation. In morphallaxis, extensive phenomena of rearrangement/recycling from differentiated tissues take place, and no blastema is formed. Thus, zebrafish regeneration adopts an epimorphosis mechanism, while starfish regeneration is an intermediate example and exhibits a transitional mechanism with overlapping morphallaxis and epimorphosis^23^.

We created *in vitro* and *in vivo* wound-healing systems to test the regenerative therapy potential of starfish extract fractions. The present study identifies NHFs as a mix of bioactive compounds to promote fibroblast-characteristic blastema formation and vascularization. NHFs cells display fibroblast-like phenotypes, active cell proliferation, and migration, while MWFs contain cytotoxic compounds, which result in cell death and cellular transformation. Corresponding to the phenotypic and behavior changes, we found that NHFs preferentially activated blastema characteristic genes (*flk1, sox9a, sox9b, pax3a*, and *foxi1*), and proteasome and autophagy genes (*psmc6, psmd12*, and *gabarapl2*), while MWFs highly upregulated *caps3a* and inhibited blastema characteristic genes (*flk1, sox9a, sox9b*, and *foxi1*; Fig. 4).

**Figure 6:**
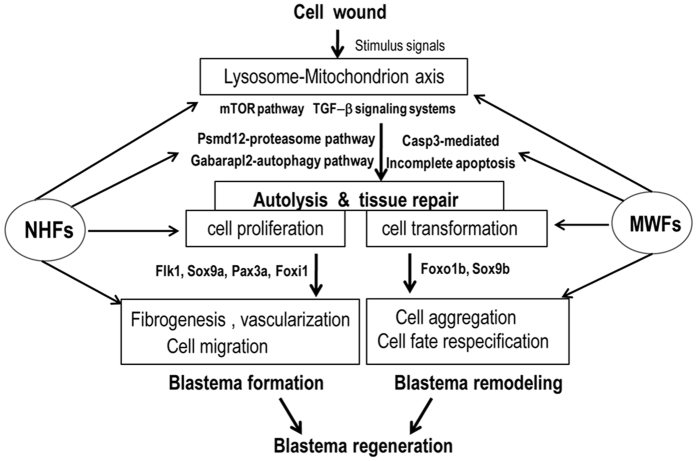
A hypothesized model of NHFs and MWFs acting in blastema regeneration. Wounding stimulates autolysis and tissue repair *via* lysosome and mitochondrion axis mechanisms, followed by blastema formation and remodeling. During the regenerative processes, TGF-β signaling systems modulate the autophagy, proteasome, and apoptosis pathways to control cell phenotypes, including cell proliferation, migration, and transformation. With the involvement of tissue-specific factors, wound repairs go through blastema formation and tissue remodeling. NHFs and MWFs, as a cocktail of multiple bioactive materials, act at the multiple regenerative steps in different ways. Generally, NHFs promote proteasome-autophagy toward blastema formation, while MWFs inducein complete apoptosis to orchestrate blastema remodeling.

The ubiquitin-proteasome system and the autophagy system are two of the most important protein degradation machineries involved in cell death, cell survival, and cell fate conversion during embryonic development and adult tissue regeneration^24^,^25^. During zebrafish caudal fin regeneration, autophagy is activated in the blastema, while inactivation of autophagy stimulates mesenchymal blastema cells to undergo apoptosis^26^. Our data suggest an active role for MWFs cytotoxicity in promoting cell transformation and preventing excessive fibroblast proliferation. The anti-proliferative effect of any agent/extract has two possible outcomes. When the extract stops or delays cell division, it said to be cytostatic. The cytostatic activity can be reversed, thereby allowing a cell to undergo cellular remodeling. Increased cell clumping and detachment signaled phenotypic changes in MWFs cells. Different from the apoptotic agent (GCV) that stimulated PAC_2_ cell overgrowth, MWFs appeared to induce high expression of *casp3a*, inhibit cell proliferation, and promote cell phenotypic changes. Since apoptotic caspases (like *caspase3*) could induce cell proliferation and nuclear reprogramming^27^,^28^, this pathway could be activated for regeneration and cancer^29^. We found that starfish extracts may activate the TGF-β signaling system, and modulate apoptosis and autophagy to control cell fate (Fig. 6).

In addition, we found that starfish biomaterials possess certain chemotaxis activities to modulate cell behaviors. Both starfish blastema-like structures and zebrafish blastema are formed at the interface between wound epidermis and blood vessels/water canal, and display coordinated cell polarization and collective migration, to some extent. This finding suggests that certain chemo-attractant molecules from wound epidermis and blood vessels or the coelomic canal direct or attract the migration of randomly migrating cells and the cells in related connective tissues.

Although peptides^30^ and molecules^31^ have been reported to mediate cell migration and aggregation, use of an *in vitro* organotypic wound-healing model has revealed a paracrine signaling network containing multiple growth factors and cytokines that orchestrate cell behavioral and phenotypic changes during wound healing^32^,^33^. As tissue regeneration is a complicated process involving multiple types of cells and tissues, a relatively pure mixture of bioactive extracts can better establish the micro-environmental niche and deliver construct cues. Indeed, platelet-rich plasma (PRP), a growth factor cocktail containing cytokines, thrombin, and other growth factors, has been widely used in cartilage repair^34^, while a combination of hyaluronic acid and PRP has synergistic anabolic actions on cartilage regeneration^10^. Perhaps through NHFs and MWFs, the presence of multiple bioactive compounds could justify tissue regeneration and coordinately remodel the new tissue structures through an integrative mechanism (Fig. 6). Our ongoing identifications and structure elucidations of active compounds will help us to learn which specific compounds exist within the NHFs and MWFs, and how to optimize the synergistic actions for scarless tissue regeneration.

## Additional Information

**How to cite this article**: Dai, Y. *et al*. Tissue Extract Fractions from Starfish Undergoing Regeneration Promote Wound Healing and Lower Jaw Blastema Regeneration of Zebrafish. *Sci. Rep.* **6**, 38693; doi: 10.1038/srep38693 (2016).

**Publisher's note:** Springer Nature remains neutral with regard to jurisdictional claims in published maps and institutional affiliations.

## Supplementary Material

Supplementary Data
