# Supplementary material for: Tissue Extract Fractions from Starfish Undergoing Regeneration Promote Wound Healing and Lower Jaw Blastema Regeneration of Zebrafish
Source: Sci Rep. 2016 Dec 15;6:38693. doi: 10.1038/srep38693 (PMC5156902; doi:10.1038/srep38693)
Supplement: Supplementary Data [file srep38693-s1.pdf]

Supplementary Information

Tissue Extract Fractions from Starfish Undergoing Regeneration Promote Wound Healing and Lower Jaw Blastema Regeneration of Zebrafish

Yancen Dai[1, $], Nagarajan Prithiviraj[2, $], Jianhong Gan[3, 4], Xin A. Zhang[5], Jizhou Yan[1, 2]*

1. Institute for Marine Biosystem and Neurosciences, Department of Biology, College of Fisheries and Life Sciences, 2. Key Laboratory of Exploration and Utilization of Aquatic Genetic Resources, Ministry of Education, 3. College of Food and Science, Shanghai Ocean University, Shanghai 201306, China. 4. Key Laboratory of Marine Biopharmaceuticals, Renji Hospital, Shanghai Jiaotong University School of Medicine, Shanghai, China. 5. Stephenson Cancer Center and Department of Physiology, The University of Oklahoma Health Sciences Center, OK 73104, USA.

$ The authors are regarded as joint First Authors

* To whom correspondence should be addressed. Tel: 862161900488; E-mail: jyan2@shou.edu.cn.

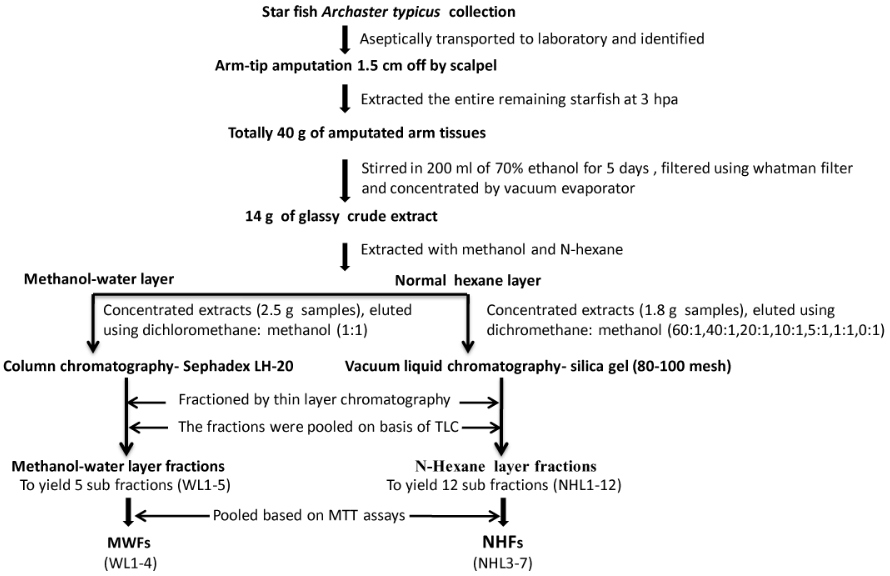

Fig. S1. Extraction and fractionation process of *A. typicus* starfish after arm-tip amputation.

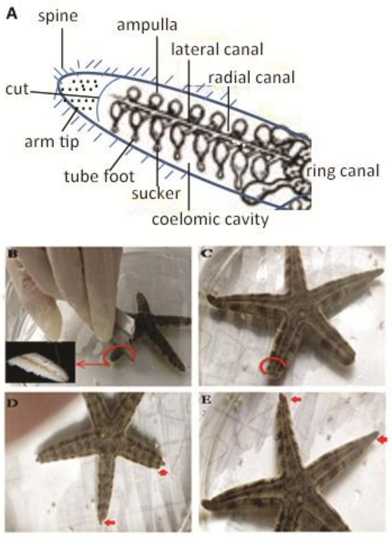

Fig.S2. Observation of *Archaster typicus* arm regeneration after amputation. (A) Water-vascular system of starfish arm.(B) Starfish arm amputated with a scalpel. (C) A small outgrowth of tissue was discernible at the end of most stumps by 6days post-amputation. (D) Photo of an arm stump on the 18th day after amputation, showing the newly formed arm-tip. (E) Photo of an arm stump on the 53rd day after amputation, showing the fully regenerated arm.

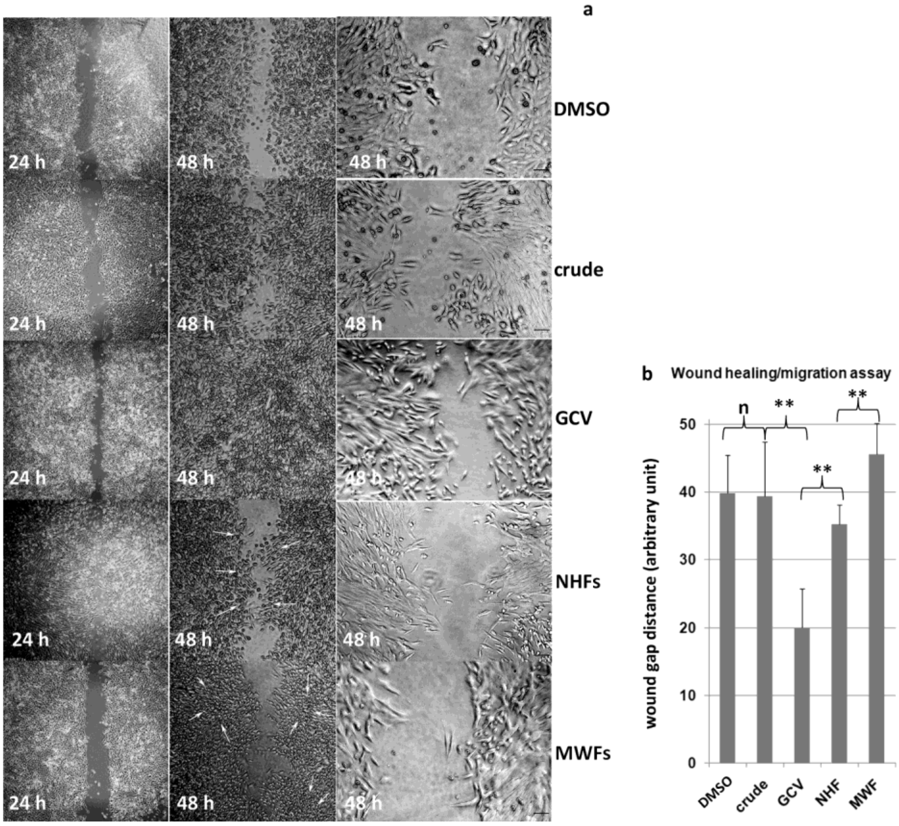

Fig.S3. Effect of *A. typicus*-extracted biomaterials on PAC_2 cell phenotype and behavior. Pac_2 cells at 80% confluence were treated with control (DMSO), or the same volume of crude extract (100 *μg/ml*), NHFs (100 *μg/ml*), MWFs (100 *μg/ml*), or ganciclovir (GCV,1uM), for 0-48 h. Migration was assessed by wound-healing assay. A wound gap was created by scratched with 200-ulpipette tip. The healing of this gap by cell migration and growth toward the center of the gap was monitored and quantified. (a) Representative images showing cell growth, migration patterns, and cell morphology at the closure of the wound area. (b) Quantification of wound gap relative distance. Average wound gap distance was measured as arbitrary units using Photoshop software. The statistical significance between two interesting groups was calculated using two-tailed *t*-test: $P<0.01$ (**); $P>0.05$(n). Scale bars represent 250,100, and 50 μm (from left to right).

Supplemental table 1: Primers used in gene expression analyses

Gene name	Forward primer	Reverse primer
**Apoptosis**		
casp3(a)	TCAATCCCATGCCTTCAGATAC	TTCCTCGACAAGCCTGAATAAA
casp 8	TTCCAGATTGAGGTACGGAATG	CTCCTTGGCAGG CTTGAATA
baxa	GGACGGAATCCGCAGTTATT	CTTTCTCTTCCCTCTCGTCTTTC
bada(tv1)	CAAAGAAGAAGAGGGCAGAGAA	GGATGGCATATCCAGCGTAAA
badb(tv1)	CAGAGGAA CCTCTCGATG AATG	TCTTTGTGTCTGGGCTCTTG
birc2	GGATGTC CAGCAGAGAG AAAG	GAGA AAGGCCCGAT TGTCATA
akt2	AGAGAGAGAGAGAGAGAGAGGA	CCAGTCGGAC GCAGTTAAAT A
**Autophagy**		
gabarapl2	TCCAGACAGAGTTCCTGTTATTG	CCGTCCTCGTCTTGTTCTTT
ifnphi3	GCACCCTGGAGTTCATACTAAA	GTCCAGT GCTGAATGGA AATG
atg5(tv1)	CTCTTTCCCGGACTCCATTAAG	CAGTCTGCCTACAGGTTGTAAA
**Proteasome**		
psmb5	CCTTCGATACGACTTGACCATAG	CTCCTCTACCCTTCGCTCTAA
psmc3	GGATGAGGTGGAGGTTTGTT	CCATCCTCCTCTTGGTCATTAG
psmc6	GTATGTGACCCAGGAGGATTTC	TGAAGTCGCTTGATGGTAGTG
psmd12	CTGCT TCGGATATGG TGTCTAC	AGTCTGGCACGTTCAATCTCC
psme3	TACTGACGCCCTGTACTAAGA	TACACTGCCCGCTAAGAAAC
**TGF-beta**		
tgfbr2	CGCGGAGTCGATGGAAATAA	TGCAGGAGCAGATATGGAAAG
bmpr1aa	CCGATGATGCCAAGAACAATAC	CCTCTCTGTCTGCCACTTATAC

bmpr1ba	GTAGCTTCATCCTCACCTTCATC	CATCTTCCCATCCACACTTCTC
rhoaa	CTTCGGCTACCTTGAGTGTTC	GGGCTGTTTCTTCCCTCTATTT
rhoab	CAGGGAACTGACCAAGATGAA	GGGAGGTGTATGACTGTGTAAAT
rhoac	TCTTGGACAGATGGTGTGTATG	GTACAGCCCAGAATCCTTTGA
smad3a	GGCCGTTAAGAGTCTAGTGAAG	CGCTGATAGTGGTAGGGATTG
smad9	AGCACTCTCTCTCACTCTCTT	ACCTGCCTACTAGCACATTTC
mycb	GACAACGAGGACGAGGATTT	GAGAAACCGCTCCACATACA
**mTOR**		
eif4ea	CAGATGGGCTCTTTGGTTCT	CCCGCTTGTTTCTCTCATCT
eif4bb	CGGGACAAAGATCCCAGAATAG	AATGGGCCAGTTTCAGGAATA
hif1ab	CCAGCGTAAGAGGAAGCTAAA	AGTGTCCGCAGTTGCTAAA
figf	GGACTGGAAGTTGTGGAGATG	GCAGGTAACTCCCTCTTTGTT
rheb	GGTCAGTTTGTCGACTCCTATG	CTCCATGTGCAGGTCTTTCT
**Tissue-specific transcription factor**		
foxi1	AGGCCATCTCAACACCAACAGACT	CCAAAGTCCACACACACACTGCAT
pax3a	AGTTCCTCATCAGCCGCAATCTGA	TGTACTGGTAAGACGCCACATGG
sox9a	AGCACATCAGCTACGGTTCCTTCA	TCTGACCTCCAGCATGGGTGTAAT
**Reference control**		
beta actin	GAACCCCAAAGCCAACAGG	GGCATACAGGGACAGCACAG
